# Financial Relief and Health Effects of Urban–Rural Health Insurance Integration on Older Rural Adults: A Causal Analysis of Age-Based Heterogeneity

**DOI:** 10.3390/healthcare14121780

**Published:** 2026-06-19

**Authors:** Sirui Li, Xiangdong Liu, Xi Wang, Shufang Zhao

**Affiliations:** Faculty of Humanities and Social Sciences, Macao Polytechnic University, Macao 999078, China

**Keywords:** urban–rural health insurance integration, medical consumption upgrading, difference-in-differences, health inequality, CHARLS

## Abstract

**Highlights:**

**What are the main findings?**

- The integration of urban–rural resident medical insurance reduced out-of-pocket health expenditure among rural older households by approximately 5.6% (*p* = 0.034), accompanied by a 1.9 percentage-point decline in the probability of catastrophic health expenditure.
- Physical health (ADL impairment score) and mental health (CES-D depression score) improved simultaneously after integration, with no significant increase in outpatient or inpatient utilization—ruling out supplier-induced demand and moral hazard as driving mechanisms.
- Financial protection was broadly distributed across educational groups, but was absent among adults aged 75 and above, revealing an age-based boundary in the policy’s benefit allocation.

**What are the implications of the main findings?**

- The integration achieved “pure financial protection”—reducing financial risk without inducing overuse of medical services—demonstrating that pooling-level upgrades can strengthen the insurance function without fueling expenditure inflation.
- The absence of a demand surge suggests that supply-side constraints in rural China—rather than moral hazard—remain the binding limit on healthcare utilization, underscoring the need to pair insurance expansion with improvements in primary care capacity.
- The exclusion of the oldest-old from financial relief highlights a structural gap: as health expenditures shift toward long-term care beyond the insurance reimbursement catalog, basic medical insurance alone cannot meet the needs of an aging population. Accelerating the integration of long-term care insurance (LTCI) with basic medical insurance is essential to close this protection gap.

**Abstract:**

Objective: To evaluate the impact of urban–rural health insurance integration on the health outcomes and financial burden of rural older adults. Methods: Utilizing panel data from the China Health and Retirement Longitudinal Study (CHARLS) spanning 2013 to 2018, we employed a staggered difference-in-differences model coupled with propensity score matching (PSM-DID) for rigorous causal identification. Results: The policy significantly reduced out-of-pocket medical expenditures for rural households by approximately 5.6% (*p* = 0.034). Concurrently, significant improvements were observed in both physical health (a 0.092-point reduction in ADL impairment scores) and mental health (a 0.725-point reduction in CES-D depression scores). Mechanism analyses revealed that the integration did not significantly increase the probability of outpatient or inpatient visits—thereby ruling out supplier-induced demand and moral hazard—while effectively reducing the incidence of catastrophic health expenditure by 1.9% (*p* = 0.004). Heterogeneity analyses indicated that while the financial relief was universally distributed across varying educational levels, the policy dividends were predominantly captured by the younger-old demographic. Notably, the reduction in financial burden was not statistically significant for the oldest-old cohort (aged 75 and older). Conclusions: The urban–rural health insurance integration has achieved a dual dividend of financial protection and health enhancement without triggering the overutilization of medical services. Nevertheless, the unmet care expenses for older adults with severe disabilities underscore the urgent necessity for a secondary safety net, such as long-term care insurance.

## 1. Introduction

China’s aging process is characterized by the coexistence of “growing old before growing rich” and an “urban-rural inversion” [1,2]. Data from the Seventh National Population Census show that the proportion of the elderly population in rural areas is significantly higher than in urban areas, and rural seniors generally face the dual pressures of low pension levels and heavy medical expenses, making them vulnerable to falling into the health poverty trap [3]. To bridge the institutional gap in healthcare coverage, the reform to integrate the basic medical insurance systems for urban and rural residents was fully launched in 2016. This reform consolidated the NCMS with the urban resident basic medical insurance (URBMI) into a unified URRBMI system, standardizing funding criteria, reimbursement benefits, and drug formularies, with coverage extending to over 1 billion insured individuals. This institutional transformation provides a rare quasi-natural experiment window for evaluating the health outcomes of universal public medical insurance.

While existing research has examined the effects of health insurance integration, further work is needed in the following areas. First, most studies have focused on intermediate outcomes such as reimbursement rates and the level of care sought [4,5]. Health is not merely about curing diseases but also encompasses the maintenance of daily living functions and psychological well-being [6]; yet, causal evaluations targeting ultimate outcomes such as physical and mental health remain scarce. Furthermore, few studies have effectively disentangled inherent urban-rural health trends to perform causal identification of the pure net effect of the policy. Second, the net effect of integration on household out-of-pocket (OOP) expenditures faces a theoretical tension: while higher reimbursement rates reduce OOP expenditures [7], the behavioral response of releasing pent-up medical demand may increase total expenditures [8]. Which of these forces prevails depends on the characteristics of existing rural healthcare demand and supply-side conditions; academia has yet to provide a clear answer. Third, the fairness of the distribution of policy benefits, particularly whether vulnerable groups such as the elderly and those with disabilities are effectively covered by the system, still lacks sufficient empirical testing [9].

Based on this, the objective of this study is to systematically evaluate the true causal net effects of the urban—rural health insurance integration on the multidimensional health and economic burden of older adults in rural areas, and to analyze the distribution patterns of policy benefits across different socioeconomic groups. The study utilizes panel data from the China Health and Retirement Longitudinal Study (CHARLS) for the period 2013–2018. The time window was defined based on quasi-natural experiment logic: the period around 2016 marked the shock phase of concentrated policy implementation, while the COVID-19 pandemic disrupted healthcare service data after 2019. Consequently, the years 2013–2018 constitute a relatively ideal and less constrained observation window for assessing the policy’s pure effects prior to the pandemic. The causal parameters derived from this window period can provide practical, cross-economic-cycle guidance for policymakers in the present and future (2026 and beyond) to deepen healthcare system reforms and improve long-term mechanisms for preventing medically induced poverty. The subsequent structure of this paper is as follows: Section 2 presents the literature review and hypotheses; Section 3 introduces the model and data; Section 4 presents the empirical results; Section 5 discusses the findings; and the paper concludes with recommendations.

## 2. Literature Review and Hypotheses

### 2.1. Literature Review

#### 2.1.1. Research on Health Inequality and Multidimensional Health Among Older Adults in Rural Areas

Health is viewed as a multidimensional capability encompassing physical, psychological, and social well-being [10]. Both the World Health Organization and the theory of social determinants of health indicate that health inequality stems from disparities in socioeconomic status, educational resources, and institutional arrangements [11]. Based on Sen’s capability approach, health measurement has shifted from single biomedical indicators to comprehensive multidimensional assessments [12]. The multidimensional poverty measurement method proposed by Alkire & Foster provides methodological support for constructing a multidimensional health deprivation index [13]. Within China’s urban-rural dichotomy, rural older adults generally face disadvantages in terms of daily living capabilities, depressive symptoms, and self-rated health [14]. Existing research largely remains at the level of static comparisons and lacks causal testing of the dynamic mechanisms underlying multidimensional health changes under policy interventions.

#### 2.1.2. Research on Health Insurance and Health Outcomes

As a core institution for risk pooling, health insurance theoretically functions through two channels: financial protection [15,16] and price subsidies [17,18,19]. However, this process is accompanied by theoretical tensions regarding demand release and moral hazard. International empirical findings exhibit significant heterogeneity: in the Oregon Health Insurance Experiment, health insurance significantly improved mental health but had no significant impact on physiological indicators (such as hypertension) in the short term, demonstrating an asymmetric effect characterized by significant mental health benefits and lagging physiological improvements [20,21]. Experiences in developing countries, such as Mexico’s Seguro Popular, indicate that health improvements are often constrained by the quality of the supply side [22,23]. In contrast, the Chinese context is more unique: constrained by both the household registration system and imbalances in the allocation of healthcare resources, rural residents face a structural contradiction between insufficient payment capacity and a shortage of high-quality care, raising questions about the applicability of Western theories [21,24,25].

#### 2.1.3. Health Outcomes of China’s Urban–Rural Health Insurance Integration Policy

The existing literature has examined the health outcomes of China’s urban–rural health insurance integration from multiple perspectives. In terms of service utilization, studies have found that integration increased reimbursement rates for outpatient care and inpatient hospitalization among rural residents [26,27] and shifted the level of care toward county-level and higher medical institutions [28,29]. Regarding costs, some studies have examined the pathways through which the integration affects total healthcare expenditures. While these studies provide important evidence on intermediate outcomes, further research is needed in three key areas. First, the evidence chain linking intermediate outcomes to ultimate health outcomes remains incomplete. Existing research has largely focused on service utilization and cost indicators, while systematic assessments of ultimate health indicators—such as activities of daily living (ADL) and depressive symptoms—remain insufficient [30]. Second, the rigor of causal identification needs improvement. Some studies have not adequately addressed the issue of policy endogeneity, and when applying multi-period DID, they have overlooked the estimation biases that may result from heterogeneous treatment effects [31]. Third, the heterogeneity of vulnerable groups is overlooked. Existing analyses are largely based on average effects and fail to reveal the differential distribution of policy benefits among vulnerable groups such as low-income and elderly populations [32,33].

### 2.2. Research Hypotheses

Based on Grossman’s (1972) health production function theory [34], health insurance influences individuals’ health investment behavior by altering the relative prices of healthcare services. The urban–rural health insurance integration has standardized benefits across urban and rural areas, effectively reducing the shadow prices of healthcare services faced by rural residents. In terms of health outcomes, the theoretical logic is relatively clear. More accessible healthcare services facilitate timely disease intervention and delay the natural decline in physical functioning (ADL) [35]. At the same time, the improved expectations regarding future risk mitigation and the sense of identity derived from national health coverage status help alleviate psychological stress and depressive symptoms (CES-D) [36,37]. Based on this, we propose Hypothesis H1:

**Hypothesis 1.**  *The urban–rural health insurance integration policy can improve the physical health (ADL) and mental health (CES-D) of middle-aged and older adults in rural areas.*

There are two competing theories regarding the impact of the integration of medical insurance schemes on household expenditures. On the one hand, an increase in the reimbursement rate directly reduces OOP expenditure, creating a price compensation effect; on the other hand, lower prices may trigger a concentrated release of long-suppressed demand for medical services. If the increase in medical visits resulting from this release of demand exceeds the savings from lower prices, household OOP expenditure may actually rise. Given the widespread phenomenon in rural areas of “delay treatment for minor illnesses and endure major ones without proper care” [38], the demand release effect is likely to dominate in the short term. Based on this, we propose the following competing hypotheses:

**Hypothesis 2a.**  *The price compensation effect prevails, and the integration policy will significantly reduce rural households’ OOP medical expenditure.*

**Hypothesis 2b.**  *The demand release effect prevails, and the integration policy will cause rural households’ OOP medical expenditure to rise rather than fall.*

Regarding the dimension of heterogeneity in distribution, there are two theoretical expectations. Based on the pent-up demand hypothesis, low-income and elderly groups have accumulated more unmet demand and are more sensitive to price changes, potentially exhibiting a stronger policy response [39]. After the integration of medical insurance systems significantly raises the benefit ceiling, this group becomes more sensitive to price changes, experiences a greater release of pent-up demand, and may see a higher increase in total medical expenses. However, Fundamental Cause Theory suggests a different conclusion: even if price barriers are eliminated, the disadvantaged groups’ limitations in accessing information and converting resources may weaken their actual benefits [40]. Furthermore, long-term care costs a significant component of the healthcare needs of the elderly, is often excluded from the medical insurance reimbursement list, thereby weakening the financial protection provided by basic medical insurance to this group. Based on this, Hypothesis H3 is proposed:

**Hypothesis 3a.**  *The impact of the integration of medical insurance schemes is more pronounced among groups with low socioeconomic status and the elderly.*

**Hypothesis 3b.**  *Due to limitations in resource conversion capabilities and the structure of care expenditures, the benefits derived from the integration by the elderly and vulnerable groups are relatively limited.*

## 3. Empirical Modeling and Data Sources

### 3.1. Model Specification

The phased integration of urban and rural resident medical insurance across different provinces provides a quasi-natural experiment setting for evaluating the policy’s effectiveness. Drawing on the methodology of Beck et al. (2010), this paper employs a staggered DID model (TWFE-DID) for causal identification [41]. The baseline regression model is specified as shown in Equation (1):

(1) $$Y_{\mathrm{ipt}}\;=\;\alpha \;+\;\beta \left({\mathrm{Treat}}_{i}{\;\times \;\mathrm{Post}}_{\mathrm{pt}}\right){\;+\;X}_{\mathrm{ipt}}^{\prime }{\gamma \;+\;\mu }_{i}{\;+\;\lambda }_{t}\;+\;\epsilon_{\mathrm{ipt}}$$

where $Y_{\mathrm{ipt}}$ denotes the health outcome of individual i (belonging to province p) in year t;${\;\mathrm{Treat}}_{i}$ is the dummy variable for the treatment group; ${\mathrm{Post}}_{\mathrm{pt}}$ represents the implementation status of the policy integration in province p during year t (post-implementation = 1); the coefficient of the interaction term ${\;\mathrm{Treat}}_{i}{\;\times \;\mathrm{Post}}_{\mathrm{pt}}$ is the average treatment effect of the policy. Control variables $X_{\mathrm{ipt}}^{\prime }$ include demographic, socioeconomic, and health-behavior characteristics; $\mu_{i}{\;\mathrm{and}\;\lambda }_{t}$ are the individual and year fixed effects$;\;\epsilon_{\mathrm{ipt}}$ is the random disturbance term. Standard errors are clustered at the provincial level.

Recent methodological research has shown that when dealing with overlapping treatment periods and heterogeneous treatment effects, the two-way fixed effects estimator can be decomposed into a weighted average of multiple 2 × 2 comparisons. In particular, the comparison where “the earlier treatment group serves as the treatment group and the later treatment group serves as the control group” may introduce negative weights, causing the estimator to deviate from the true average treatment effect [42,43]. In the context of this study, the CHARLS data cover only the 2013, 2015, and 2018 survey years, and policy shocks are highly concentrated in the 2016–2018 window. Given the limited variation in treatment timing, the risk of severe bias arising from heterogeneous treatment effects is relatively manageable. Nevertheless, the subsequent robustness analysis section conducts sensitivity assessments of potential biases by excluding samples from provinces with delayed policy implementation, setting fictitious policy implementation dates, and changing identification strategies. To confirm the robustness of the empirical results, this study further introduces a propensity score matching (PSM)—differ-in-differences (DID) model. The matching process employs a 1:1 nearest-neighbor matching algorithm with a caliper value of 0.05, and matching is strictly based on baseline data from 2013—prior to the policy intervention—to ensure that the balance of covariates is not affected by policy interference.

### 3.2. Variable Measures

#### 3.2.1. Dependent Variable

Based on the World Health Organization’s multidimensional definition of health, we developed an evaluation system encompassing physical health, mental health, and economic burden (see Table 1) [44]. Physical health was measured using the ADL scale to capture the policy’s effect on maintaining physical function among older adults [45]. Mental health is assessed using the 10-item Center for epidemiologic studies depression scale (CES-D). Higher scores on this scale indicate more severe depressive symptoms; in this sample, the Cronbach’s α coefficient was 0.840, indicating good internal consistency [46]. Economic burden was measured using the natural logarithm of annual OOP medical expenditures. Out-of-pocket expenditures were calculated by summing total expenditures on outpatient care, inpatient care, and self-purchased medications, minus reimbursements from various health insurance plans [47].

#### 3.2.2. Core Explanatory Variables

The core explanatory variables were constructed based on the timing of policy implementation and the respondents’ household registration status. The temporal dimension (Post) defines the year of the policy shock based on the State Council’s “Opinions on Integrating the Urban and Rural Resident Basic Medical Insurance Systems” and the actual implementation progress in each province [48]. Among the 28 provinces covered by the CHARLS dataset, 25 completed the substantive integration by 2017 or earlier, while Beijing, Jiangsu, and Gansu did not complete the integration until 2018; the model thus defines the policy implementation status for each province accordingly [49]. The definition of the treatment group (Treat) is based on an individual’s household registration status. Prior to integration, residents with agricultural hukou were enrolled in the new cooperative medical scheme (NCMS), while urban non-working residents were enrolled in the urban resident basic medical insurance; the two systems operated separately and in parallel. Household registration status (particularly for middle-aged and older adults aged 45 and above) exhibits strong temporal stability and exogeneity. Grouping based on household registration aligns with the intention-to-treat (ITT) framework [50]. It helps avoid sample selection bias that might arise from categorization based on self-reported insurance types.

#### 3.2.3. Mechanism Variables

To further analyze the micro-level sources of medical cost growth and the risk-mitigating effectiveness of policies, this study selected two key indicators based on the theory of health insurance mechanisms [20]. First, regarding healthcare service utilization, we selected two binary variables—whether the individual visited an outpatient clinic in the past month and whether they experienced inpatient hospitalization in the past year—to examine whether, following the relaxation of budget constraints, rural residents’ healthcare-seeking behavior exhibited low-level quantitative expansion or high-intensity intensification. Second, regarding protection against major illness risks, we constructed a dummy variable for catastrophic health expenditure (CHE) based on the method proposed by Wagstaff & Lindelow [51]. This indicator is defined as whether a household’s out-of-pocket medical expenditures exceed 40% of total household consumption (yes = 1, no = 0). It is used to verify whether the integration of health insurance schemes, while unleashing demand for high-end medical services, has effectively fulfilled its safety-net function in preventing medically induced poverty [47].

#### 3.2.4. Control Variables

Following the basic assumptions of Grossman’s health production function, the model incorporates three categories of control variables to effectively isolate the effects of confounding factors [34]. These include demographic characteristics such as age, gender, and marital status; socioeconomic status characteristics such as years of education and employment status; and health behavior variables such as smoking and alcohol consumption. It should be noted that health shocks may simultaneously affect both medical expenditures and labor capacity, thereby creating a bidirectional causal relationship with household income; treating household income as a control variable may introduce the problem of “bad control” [52]. In light of this, the subsequent robustness analysis section will report regression results after excluding the household income variable to assess the sensitivity of the estimates to this variable. Descriptive statistics for all control variables are presented in Table 2. Standard errors are subject to province-level clustering to account for within-group correlation.

### 3.3. Data Sources and Processing

The micro-level empirical data used in this study were drawn from the 2013, 2015, and 2018 waves of the CHARLS. Data on the key policy implementation dates were manually compiled from official documents such as the “Implementation Plan for the Integration of Urban and Rural Resident Medical Insurance” issued by provincial governments. Data cleaning followed the following logic: First, we screened for middle-aged and older adults who participated in all three waves and were aged 45 or older at baseline (2013) to construct a balanced panel. Second, we performed inter-period harmonization on core variables and used multiple imputation methods to impute missing values to maintain data stability. Finally, we matched the micro-level individual data with provincial policy timelines and macro-level control variables. Following these procedures, a final dataset of 48,732 observations was obtained.

## 4. Results

### 4.1. Descriptive Statistics

Table 2 presents descriptive statistics for the full sample and the results of tests for differences between urban and rural groups. The treatment group (those with agricultural hukou) accounts for approximately 77.7% of the sample, which is higher than the urbanization rate of the permanent resident population reported by the National Bureau of Statistics. This discrepancy arises because the CHARLS survey is limited to individuals aged 45 and older, and the retention rate of agricultural hukou is significantly higher among this age group than across all age groups [53]. Since eligibility for the Urban Resident Basic Medical Insurance prior to the integration was tied to household registration status, defining the treatment group based on agricultural hukou allows for the recreation of the actual constraints prior to the policy shock, consistent with the ITT principle.

A *t*-test comparing mean scores across groups revealed disparities between urban and rural populations across multiple dimensions prior to the integration. In terms of health outcomes, the rural elderly population faces dual vulnerabilities—both physical and psychological: their level of physical impairment (ADL score of 0.459) was higher than that of the urban population (0.317), and their level of psychological depression (CES-D score of 5.542) also showed a statistically significant disadvantage (T = −8.948, *p* < 0.001), confirming the cumulative effects of unequal distribution of urban-rural medical resources and differences in living environments on vulnerable groups. In terms of economic characteristics and socioeconomic status, the rural group’s average years of education (4.34 years) lagged behind that of the urban group (7.59 years). Yet, their proportion of the workforce (42.4%) exceeded that of the urban group (10.2%). This mismatch of low education and high labor participation reflects the weakness of the rural pension security system, making labor a necessary choice for sustaining livelihoods. In terms of healthcare expenditure, the logarithm of annual OOP expenditure for the rural group (0.362) was lower than that of the urban group (0.431) prior to the integration of the medical insurance systems. Combined with the relatively poorer baseline health status of the rural population, the coexistence of high health risks and low healthcare expenditures reflects the limitations in protection resulting from institutional disparities prior to the integration. Structural differences in baseline characteristics indicate that simple cross-sectional comparisons are prone to being distorted by heterogeneity among sample groups, highlighting the necessity of using a difference-in-differences model to control for pre-existing differences and identify the net effect.

### 4.2. Baseline Effect Analysis

Table 3 presents the baseline regression results regarding the impact of the urban–rural health insurance integration on health outcomes and financial burdens among the rural elderly population. After controlling for micro-level individual characteristics as well as two-way fixed effects for province and year, the empirical results reveal the dual dividend of the integration policy: financial safety nets and improvements in health.

In terms of the economic burden, the implementation of the policy has alleviated the pressure of medical expenses on rural households. As shown in Column (3), the estimated coefficient of the core explanatory variable Did for the logarithm of OOP medical expenditure (Ln *OOP*) is −0.058 (*p* = 0.034). After retransformation from the log form, this implies that the integration of medical insurance systems has reduced the absolute OOP medical expenditure of rural respondents by approximately 5.6%. The data confirm that by raising the pooling level, standardizing reimbursement rates, and expanding the scope of coverage, the integration of medical insurance successfully broke through the coverage bottleneck caused by the limited fund pool under the original New Rural Cooperative Medical Scheme (NRCMS), effectively fulfilling the core function of medical insurance in dispersing financial risks. At the same time, policy benefits have also become evident in terms of health outcomes. Since the measures of physical health (ADL) and mental health (CES-D) used in this study are both negative-scaled indicators, the results in columns (1) and (2) of Table 3 are positive. The coefficient for ADL is −0.092 (*p* = 0.003), and the coefficient for CES-D is −0.725 (*p* < 0.001); both are negative. This indicates that as financial constraints on healthcare were eased, the rural elderly population gained access to more timely and higher-level healthcare services, thereby reducing the degree of functional impairment and alleviating psychological depression. These findings not only validate the effectiveness of health insurance system integration in narrowing the urban-rural gap in health and welfare but also support the study’s hypothesis H1, which posits that the policy of urban–rural health insurance integration can simultaneously reduce economic burdens and improve health outcomes.

### 4.3. Testing for Parallel Trends and an Examination of Endogeneity

To test the identification assumptions of the double-difference model and capture the dynamic characteristics of policy, this paper employs the event study approach, using 2015 as the base year to construct interaction terms for regression analysis (see Equation (2) for the model specification).

(2) $$Y_{\mathrm{it}}{=\beta }_{0}{+\beta }_{1}\left({\mathrm{Treat}}_{i}{\;\times \;\mathrm{Year}}_{2013}\right){+\beta }_{2}\left({\mathrm{Treat}}_{i}{\;\times \;\mathrm{Year}}_{2018}\right)+\mathrm{Controls}+\mathrm{FE}+\epsilon_{\mathrm{it}}$$

where $Y_{\mathrm{it}}$ represents the dependent variable (ADL, CES-D, or LnOOP), given that the national-level integration policy, the “Opinions,” was issued in 2016, we set the most recent observation year prior to policy implementation (2015) as the base period (systematic multicollinearity in the model was omitted). We focus on the coefficients of the pre-policy interaction term ${\mathrm{Treat}}_{i}{\;\times \;\mathrm{Year}}_{2013}$ and the post-policy interaction term ${\mathrm{Treat}}_{i}{\;\times \;\mathrm{Year}}_{2018}$. The former $\beta_{1}$ is used to test the parallel trends hypothesis, while the latter $\beta_{2}$ is used to capture the dynamic policy effects.

**Figure 1:**
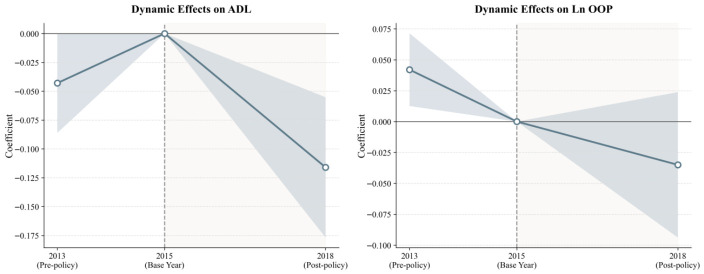
Test for Parallel Trends. Note: Solid dots show the dynamic treatment effect coefficients for each year, the shaded ribbon represents the 95% confidence interval, the horizontal solid line is the zero-effect baseline, the vertical dashed line marks the policy implementation year (2015), and the light gray area indicates the post-policy period.

The results in Table 4 indicate that the standard difference-in-differences model faces a pre-existing challenge related to parallel trends in this sample. In the economic burden dimension, the coefficient of the pre-policy interaction term (${\mathrm{Treat}\;\times \;\mathrm{Year}}_{2013}$) is 0.042 (*p* = 0.009). Prior to the full implementation of the policy in 2016, the trajectories of OOP medical expenditure for the treatment group and the control group were not parallel (see Figure 1), indicating structural differences in baseline levels between the two groups. Similarly, in the health outcomes dimension, the pre-policy interaction terms for physical and mental health showed marginally significant (−0.043, *p* = 0.061) and highly significant (−0.288, *p* = 0.015) coefficients, respectively. These pre-policy differences reveal a core endogeneity dilemma in causal identification: the rate of health decline and healthcare-seeking behavior among rural populations are constrained by long-standing socioeconomic disadvantages, resulting in development trajectories that differ from those of urban residents. Therefore, although the baseline regression captures positive signals, the baseline model confounds these with time-varying unobservables inherent to urban-rural heterogeneity.

**Figure 2:**
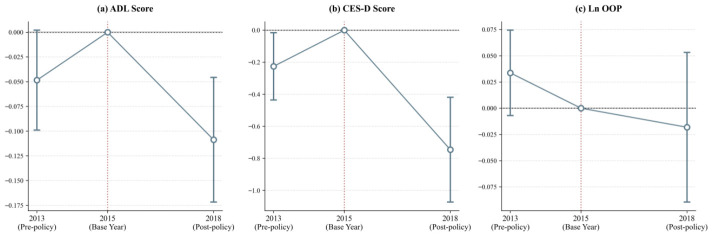
Collated results of the post-matching test for parallel trends. Note: Solid dots show the dynamic treatment effect coefficients for each year, error bars represent 95% confidence intervals, the horizontal solid line is the zero-effect baseline, and the red vertical dashed line marks the policy base year (2015).

To more rigorously assess the effectiveness of the PSM-DID identification strategy, Table 5 and Figure 2 present the event study estimates for the matched sample. After controlling for observable characteristic differences, the pre-policy coefficient for out-of-pocket costs (Ln *OOP*) decreased to 0.034 (*p* = 0.117), losing statistical significance and satisfying the parallel trends assumption.

In the health outcomes dimension, the pre-policy coefficient for mental health (CES-D) was −0.226 (*p* = 0.044), and the pre-policy coefficient for physical health (ADL) was −0.048 (*p* = 0.076). The pre-intervention differences across these two dimensions were not fully eliminated after matching; however, when comparing the relative magnitudes of the pre-intervention differences and post-intervention effects, the CES-D coefficient after the policy intervention (2018) was −0.746 (*p* < 0.01), with an absolute value approximately 3.3 times that of the pre-intervention difference (0.226); the post-intervention coefficient for ADL was −0.109 (*p* = 0.002), approximately 2.3 times the pre-intervention difference (0.048). The magnitude of the post-intervention effects was significantly greater than the baseline differences remaining after matching, and the post-intervention coefficients were consistent with the baseline regression results in terms of direction and significance, indicating that the causal identification results for the matched sample were generally robust.

### 4.4. Robustness Tests

To verify the reliability of the estimates from the baseline model, this paper conducted multiple robustness tests from various perspectives, including sample sensitivity, placebo tests, and PSM (the results are reported in Table 6).

#### 4.4.1. Sample Sensitivity and Policy Timing Tests

To rule out the influence of specific sample characteristics and differences in policy implementation timing, this study conducted two restriction tests: (1) Excluding samples from municipalities directly under the central government. Given the unique characteristics of these municipalities in terms of healthcare resource endowments and the size of their medical insurance funds, Panel A excluded samples from the four municipalities directly under the central government. Regression results show that the coefficient of Did on Ln *OOP* increased to −0.066 and was significant at the 5% level. This confirms that the conclusion regarding the reduction in financial burden is not driven by outliers in large cities and is broadly applicable nationwide. (2) Exclusion of samples with insufficient policy exposure. Given that the policy was coordinated and implemented by local governments, some provinces did not complete the integration until as late as 2018, resulting in insufficient policy exposure duration for respondents. In Panel C, after excluding the aforementioned provinces with delayed implementation, the coefficient of the DID interaction term remained stable (−0.048, *p* = 0.087). The data confirm that, after controlling for the interference of delayed implementation, the financial burden-reduction effect of health insurance integration remains valid, establishing the robustness of the policy’s long-term efficacy.

#### 4.4.2. Placebo Test and Counterfactual Tests

**Figure 3:**
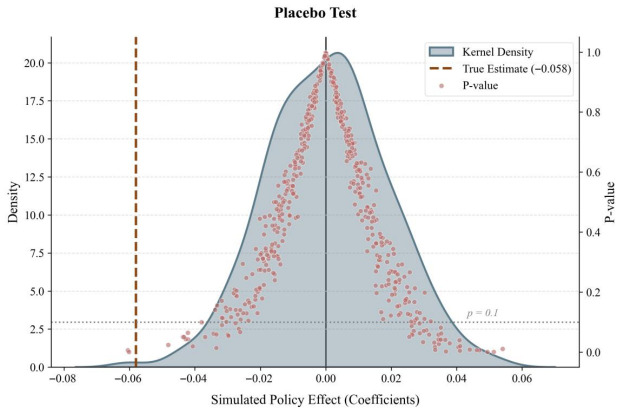
Distribution of coefficients from the placebo test.

To rule out the influence of omitted variables and pre-existing endogenous trends, this study conducted two falsification tests: (1) a counterfactual policy timing test. In Panel B, the policy implementation date was artificially moved forward to 2015, and only the actual pre-policy data (2013 and 2015) were retained in the full sample for a counterfactual regression. After excluding the years of the actual policy shock, the estimated coefficient of the dummy policy variable (Did Fake) on the economic burden was significantly negative (−0.040, *p* = 0.013). This counterfactual result, together with the findings in Section 4.3, demonstrates that spontaneous fine-tuning of the original NRCMS coverage level had already led to a certain baseline trend of reduced financial burden prior to the implementation of the national top-level policy. However, the absolute magnitude of this dummy variable coefficient (−0.040) is smaller than the long-term effect of the actual policy in the baseline model (−0.058). From the perspective of this difference in magnitude, this confirms that only the actual policy of integrating the two schemes possesses a breakthrough impact in reducing the financial burden. (2) Randomized placebo test (Figure 3). To verify whether concurrent random macroeconomic shocks caused the effect, this study generated 500 “pseudo-treatment groups” from the full sample and repeated the regression analysis. The kernel density plot shows that the simulated dummy coefficients follow a standard normal distribution with a mean of 0, and the vast majority of *p*-values are greater than 0.1. In contrast, the estimated Did coefficient (−0.058, shown by the red dashed line) deviates significantly from the core distribution range of the dummy coefficients, falling into the left-tail region. This robust spatial falsification rules out the possibility of random coincidence.

#### 4.4.3. Cross-Model Validation and Selection Bias Elimination

**Figure 4:**
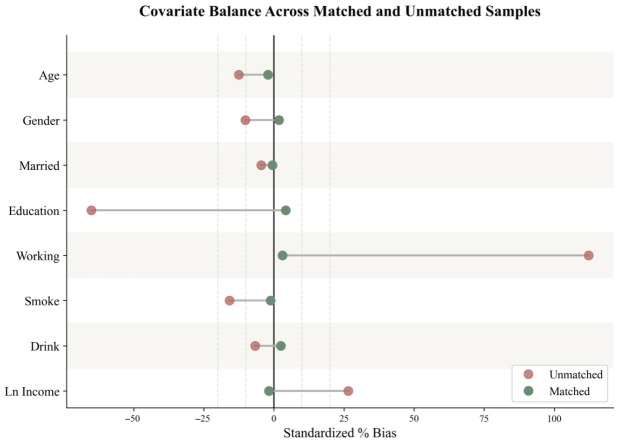
Covariate Balance Across Matched and Unmatched Samples. Note: Red dots indicate standardized bias before matching, green dots indicate bias after matching, gray lines show the reduction in bias, the solid vertical line is the zero-baseline, and dashed vertical lines represent the ±10% bias threshold.

The baseline model did not fully pass the parallel trends test. To address selection bias, this study employs PSM-DID for cross-model validation. Through 1:1 nearest-neighbor matching at the baseline period (2013), the standardized deviations of the vast majority of covariates were reduced to within 10% (Figure 4), thereby constructing a balanced panel dataset with high comparability. Table 6 (Panel D) shows that after controlling for group differences in observable characteristics, the positive effects of DID on physical health (−0.082) and mental health (−0.620) remain significant. Meanwhile, the coefficient for financial burden remained consistent in both magnitude and direction (−0.037). Although statistical significance decreased due to the loss of some samples during matching, the overall results, when combined with the aforementioned multidimensional test matrix, still corroborate the empirical conclusion that the integration of health insurance systems alleviates the financial burden of medical care.

#### 4.4.4. Test of Excluding Household Income as a Control Variable

Table 5, Panel E reports the results of a robust regression after excluding the log of household income. As shown in the table, after excluding the income variable, the estimated coefficient of the core explanatory variable Did on economic burden (Ln *OOP*) is −0.061 and is significant at the 5% level; the estimated coefficients on physical health (ADL) and mental health (CES-D) are −0.099 and −0.706, respectively, and both are significantly negative at the 1% level. Compared to the baseline model that includes household income (with corresponding coefficients of −0.058, ADL: −0.092, and CES-D: −0.725), the core explanatory variable exhibits high stability in terms of coefficient direction, magnitude, and statistical significance. The baseline estimates in this study are not sensitive to the specific settings of control variables, and potential “bad control” biases do not pose a substantial threat to the core causal inferences.

### 4.5. Mechanism Testing

To analyze the micro-level transmission mechanisms through which the integration of medical insurance systems reduces the burden of medical expenses, this paper further examines two dimensions: healthcare utilization behavior and economic risk mitigation (Table 7).

The results of the mechanism tests reflect the robust characteristics of the policy implementation process. The data in Columns (1) and (2) show that the integration did not lead to significant changes in the probability of outpatient visits or hospital stays among rural residents, indicating that the policy expansion did not result in excessive utilization of medical resources by micro-level agents. Against this backdrop, the results in Column (3) show that the probability of rural households incurring CHE decreased by 1.9% (*p* = 0.004). These findings effectively address the competitive hypothesis proposed earlier. Without significantly increasing the total volume of services in the healthcare system, the price compensation effect of the health insurance integration successfully counteracted the potential demand-release effect. Rural households did not experience the retaliatory rebound in healthcare consumption feared in Hypothesis H2b, and the empirical results support the pure financial protection pathway described in Hypothesis H2a.

### 4.6. Heterogeneity Analysis

**Figure 5:**
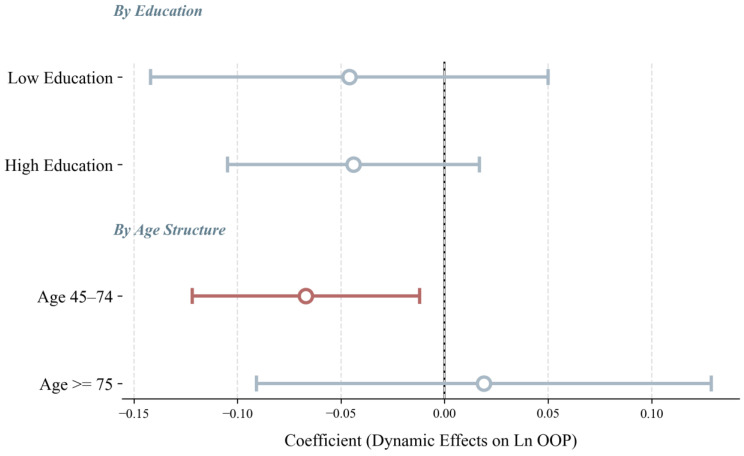
Analysis of Heterogeneity in Policy Effects. Note: terracotta red indicates significance (*p* < 0.05), light grayish blue indicates non-significance, error bars represent 95% CI, and the vertical line x = 0 denotes no effect.

The distribution of the inclusive benefits resulting from institutional integration across different groups is a key factor in determining the fairness of the policy. This paper conducts a heterogeneous regression analysis based on educational attainment and age structure (Table 8 and Figure 5).

The analysis results reveal distinct patterns in the scope of policy impact. On the one hand, regarding socioeconomic status (SES), the reduction in healthcare costs for the low-education group and the high-education group was highly consistent in magnitude (–0.046 and –0.044, respectively). The universal nature of the cost-reduction effect across different educational groups fails to support the hypothesis in H3a that individuals with absolute essential needs would exhibit a stronger behavioral response. On the other hand, intergenerational heterogeneity was significant. The policy benefits were primarily evident among middle-aged and older adults (45–74 years old, Did = −0.067, *p* = 0.024). In contrast, the limited benefits for those aged 75 and older corroborate the core logic of Hypothesis H3b. That is, universal health insurance struggles to fully penetrate the non-medical financial barriers arising from aging and disability, leading to a decline in the effectiveness of policy benefits among the most vulnerable elderly population.

## 5. Discussion

### 5.1. Synergies Between Financial Protection and Health Improvements

Empirical results show that the urban–rural health insurance integration reduced actual OOP expenditure for middle-aged and elderly rural residents (by approximately 5.6%) while simultaneously leading to improvements in indicators of physical functioning (ADL) and psychological depression (CES-D). In existing evaluations of international health insurance systems, the classic “Oregon Health Insurance Experiment” [20] observed that expanding insurance coverage can rapidly improve the psychological well-being of low-income groups. However, the short-term effects of such interventions on physiological indicators, such as functional impairment, were unclear. Compared to the asymmetric pattern observed in Western literature—where “psychological benefits are significant but physiological improvements lag”—the dual improvement effect observed in this study stems from the unique baseline constraints and intensity of policy interventions among China’s rural population. Analyzed through the lens of Grossman’s (1972) health capital theory, rural elderly populations have long been subject to high healthcare budget constraints [54], making their health investment behavior sensitive to service prices. Following the integration of the healthcare system, the elevation of the pooling level substantially reduced the relative prices of clinical treatments. This price leverage not only directly alleviated the anxiety and depression caused by medically induced poverty but also, by easing financial constraints, prompted more timely primary healthcare interventions, effectively counteracting the natural decline in physical function. These findings confirm the health spillover benefits of public health insurance beyond its role as a financial safety net, providing robust causal evidence for developing countries to narrow the urban-rural health welfare gap through institutional integration under resource-constrained conditions.

### 5.2. Mitigating Moral Hazard and Ensuring Financial Safety Nets

Empirical analysis reveals that the integration policy did not lead to a statistically significant increase in the probability of rural residents seeking outpatient or inpatient care. However, the risk of households incurring CHE decreased significantly by 1.9%. In traditional health economic discussions regarding the expansion of basic health insurance, supplier-induced demand and moral hazard are mechanisms of widespread concern in the academic community. Some previous empirical studies have argued that higher reimbursement levels stimulate overutilization of medical services, thereby driving up systemic total costs [55,56]. However, the micro-level pathways outlined by the data in this study diverge from these concerns: the stable trend in healthcare utilization rules out disorderly resource misuse, while the reduction in extreme financial risk reflects how the expansion of the fund pool has effectively alleviated the burden of severe illnesses. This phenomenon of “pure financial protection without overtreatment” is attributed to the institutional safeguards designed during China’s health insurance pooling process. While policies have increased reimbursement rates, they have retained strict deductibles and copayment mechanisms [57] and strengthened regulatory constraints on the medical insurance formulary. This ensures that newly pooled funds primarily fulfill the essential insurance function of hedging against the tail risks of severe illnesses, rather than providing excessive subsidies for minor ailments. The empirical results support the competitive hypothesis that price compensation effects prevail, confirming that through the optimization of risk-sharing mechanisms, universal health insurance can fulfill its baseline function of preventing medically induced poverty without increasing the overall financial burden.

### 5.3. Policy Benefits Fail to Penetrate Non-Medical Barriers Facing the Elderly with Disabilities

Heterogeneity analysis indicates that the reduction in medical burdens exhibits universal benefits across groups with different educational levels; however, intergenerational heterogeneity in benefits reveals implicit boundaries in institutional coverage, as no substantial relief in burdens was observed among the elderly aged 75 and older. This divergence partially deviates from and complements the classic predictions of the Fundamental Cause Theory in sociology. This theory typically posits that socioeconomic status determines why disadvantaged groups struggle to access policy benefits equitably [58]. The universal nature of the educational dimension in the research findings reflects that the integration policy demonstrates strong institutional inclusivity in process optimization and benefit disbursement, successfully overcoming barriers to information conversion. However, the limited benefits for the elderly expose the inherent blind spots in basic medical insurance, which is oriented toward clinical cure. As the aging process progresses, the core expenditure structure of the elderly gradually shifts from routine outpatient, emergency, and inpatient costs to long-term care and disability care costs that fall outside the scope of medical insurance reimbursement. Relying solely on health insurance for medical conditions cannot penetrate the non-medical financial barriers caused by physiological decline. These findings clearly highlight the limitations of the current health system reform and suggest that policy design should focus on transitioning from single-item expense reimbursement to comprehensive elderly care, providing crucial empirical support for accelerating the institutional integration of health insurance and long-term care insurance (LTCI).

### 5.4. Policy Implications

Based on the robust findings outlined above, this study offers the following policy implications: First, we should maintain and deepen the steady improvement of the pooling level of medical insurance, further narrow the de facto gap in benefits between urban and rural areas, and consolidate the basic medical insurance’s role as a safety net against medically induced poverty; Second, to address the coverage gaps for the elderly and disabled, efforts should be accelerated to pilot and expand the LTCI system in rural areas, establishing a multi-tiered, coordinated protection network comprising “basic medical insurance + critical illness insurance + LTCI” to fill the coverage vacuum for non-medical care expenses; Third, we must strengthen the refined and intelligent management of medical insurance funds. While maintaining a high level of financial protection, we should comprehensively promote reforms in medical insurance payment methods, such as DRG and DIP, to effectively prevent potential medical inflation and demand-inducing risks at the institutional source.

### 5.5. Limitations

Due to data limitations, this study also has several limitations. First, the empirical analysis is based on demand-side micro-level household data and does not examine the impact of health insurance payment reform on supplier behavior. Future research could combine hospital-level prescription and cost data to explore the synergistic effects of health insurance integration and payment reforms such as DRG/DIP. Second, due to the time span of the CHARLS panel data, the study was unable to assess the policy’s impact on longer-term health indicators such as life expectancy and the progression of chronic diseases. Third, even after Propensity Score Matching (PSM), residual differences in pre-policy trends on the mental health dimension persist between the treatment and control groups. This constitutes a constraint on causal identification; therefore, point estimates of the CES-D effect should be interpreted with caution. A more precise assessment of the magnitude of this effect could be conducted if longer-term panel data or superior matching tools become available.

## 6. Conclusions

Through a micro-level empirical examination of the urban–rural health insurance integration policy, this study reveals the dual dividend of financial safety nets and health benefits. Empirical findings show that the integration of health insurance successfully broke down existing household registration barriers and institutional fragmentation, significantly reducing OOP expenditure and the risk of CHE among the rural elderly. More importantly, this burden-reduction effect was achieved without inducing supplier-induced demand or overtreatment, demonstrating the superiority of policies focused purely on financial protection. With the alleviation of economic pressures, both the physical functioning and mental health of rural elderly improved in the short term, reflecting a leap forward in health equity. However, the distribution of these health benefits is constrained by generational boundaries, and the care burden for frail elderly individuals remains outside the scope of the current basic medical insurance framework.

## Figures and Tables

**Table 1:** Definitions and Measures of Multidimensional Health Outcomes.

Variable Dimensions	Variable Name	Variable Description and Measurement Method	Theoretical/Literature Basis
Physical Health	ADL	A total score is calculated based on the difficulty level of six basic activities (dressing, bathing, eating, getting out of bed, using the toilet, and controlling bowel/bladder functions). A higher score indicates poorer self-care ability.	Katz et al. [45]
Mental Health	CES-D	The total score is calculated based on the 10-item CES-D Depression Self-Rating Scale. A higher score indicates more severe depressive symptoms.	Radloff [46]
Financial Burden	Ln *OOP*	Sum the net out-of-pocket expenses paid by the respondent for outpatient visits, hospitalizations, and self-purchased medications (after deducting reimbursed portions).	Wagstaff [47]

**Table 2:** Descriptive Statistics for Variables and Tests for Differences Between Groups.

	Variables	Total Sample Mean (48,732)	Total Sample SD	Control Group Urban	Treatment Group Rural	Mean Difference Diff	T-Value	*p*-Value
Dependent variable	ADL score	0.428	1.080	0.317	0.459	−0.142	−12.136	*p* < 0.001
	CES-D	5.417	5.785	4.980	5.542	−0.562	−8.948	*p* < 0.001
	Ln *OOP*	0.377	0.779	0.431	0.362	0.069	8.198	*p* < 0.001
Control variables	Age	61.920	9.874	62.831	61.658	1.173	10.953	*p* < 0.001
	Gender (1 = Male)	0.480	0.500	0.526	0.466	0.059	10.937	*p* < 0.001
	Marital status (1 = Married)	0.854	0.353	0.861	0.851	0.010	2.580	*p* = 0.010
	Education	5.065	4.248	7.588	4.339	3.248	74.278	*p* < 0.001
	Working	0.352	0.478	0.102	0.424	−0.322	−64.590	*p* < 0.001
	Smoke	0.056	0.229	0.077	0.049	0.028	11.380	*p* < 0.001
	Drink	0.258	0.438	0.275	0.254	0.021	4.433	*p* < 0.001
	*Ln ⁡Income*	1.053	1.457	0.797	1.127	−0.330	−20.934	*p* < 0.001
	Outpatient Visit	0.197	0.398	0.196	0.197	−0.001	−0.310	*p* = 0.756
	Inpatient Visit	0.038	0.191	0.038	0.038	0.000	−0.001	*p* = 0.999
	CHE	0.077	0.266	0.072	0.078	−0.006	−2.078	*p* = 0.038

Note: Difference in mean values (Diff) = control group—treatment group.

**Table 3:** Baseline Regression Results on Health Outcomes and Economic Burden Following the Integration of Medical Insurance Schemes.

Variables	(1) ADL	(2) CES-D	(3) Ln *OOP*
Did (Treat × Post)	−0.092 (*p* = 0.002)	−0.725 (*p* < 0.001)	−0.058 (*p* = 0.034)
	−0.028	−0.151	−0.026
Treat	0.221 (*p* < 0.001)	0.545 (*p* = 0.001)	−0.019 (*p* = 0.195)
	−0.024	−0.139	−0.014
Post	−0.01(*p* = 0.565)	−6.269 (*p* < 0.001)	0.070 (*p* = 0.001)
	−0.017	−0.104	−0.02
Age	0.021 (*p* < 0.001)	−0.022 (*p* < 0.001)	0.003 (*p* = 0.001)
Gender	−0.019 (*p* = 0.293)	−1.106 (*p* < 0.001)	0.008 (*p* = 0.377)
Married	−0.089 (*p* < 0.001)	−0.356 (*p* < 0.001)	0.035 (*p* = 0.025)
Education	−0.022 (*p* < 0.001)	−0.011 (*p* = 0.099)	−0.001 (*p* = 0.564)
Working	−0.265 (*p* < 0.001)	0.623 (*p* < 0.001)	−0.089 (*p* < 0.001)
Smoke	0.121 (*p* < 0.001)	0.003 (*p* = 0.977)	0.008 (*p* = 0.536)
Drink	−0.117 (*p* < 0.001)	−0.008 (*p* = 0.886)	−0.132 (*p* < 0.001)
*Ln Income*	−0.014 (*p* = 0.006)	0.038 (*p* = 0.031)	−0.006 (*p* = 0.080)
Provincial fixed effects	YES	YES	YES
Fixed effects	YES	YES	YES
Observed value (Obs)	48,732	48,732	48,732
$R^{2}$	0.096	0.42	0.019

Note: The robust standard errors, calculated using provincial clusters, are listed below the coefficients. The exact *p*-values are shown in parentheses next to the coefficients. Both the dependent variables, ADL and CES-D, are negative-scaled measures; lower scores indicate better health status.

**Table 4:** Parallel Trends Test and Analysis of Dynamic Effects.

Variables	(1) ADL	(2) CES-D	(3) Ln *OOP*
Pre-policy (2013)	−0.043 (*p* = 0.061)	−0.288 (*p* = 0.015)	0.042 (*p* = 0.009)
(${\mathrm{Treat}\;\times \;\mathrm{Year}}_{2013}$)	−0.022	−0.111	−0.015
Base Year (2015)	0	0	0
(${\mathrm{Treat}\;\times \;\mathrm{Year}}_{2015}$)	(omitted)	(omitted)	(omitted)
Post-policy (2018)	−0.116 (*p* < 0.001)	−0.883 (*p* < 0.001)	−0.035 (*p* = 0.254)
(${\mathrm{Treat}\;\times \;\mathrm{Year}}_{2018}$)	−0.031	−0.169	−0.03
Control variables and fixed effects	YES	YES	YES
Observed value (Obs)	48,732	48,732	48,732
$R^{2}$	0.096	0.42	0.019

Note: The robust standard errors, calculated using provincial clusters, are listed below the coefficients. The exact *p*-values are shown in parentheses next to the coefficients. The CES-D model includes all complete data from all three phases.

**Table 5:** Post-matching Test of Parallel Trends.

Variables	(1) ADL	(2) CES-D	(3) Ln *OOP*
Pre-policy (2013)	−0.048 (*p* = 0.076)	−0.226 (*p* = 0.044)	0.034 (*p* = 0.117)
	−0.026	−0.107	−0.021
Post-policy (2018)	−0.109 (*p* = 0.002)	−0.746 (*p* < 0.001)	−0.018 (*p* = 0.621)
	−0.032	−0.167	−0.036
Treat	0.246 (*p* < 0.001)	0.643 (*p* < 0.001)	−0.050 (*p* = 0.065)
	−0.026	−0.152	−0.026
Provincial fixed effects	YES	YES	YES
Observed value	44,475	44,475	44,475
$R^{2}$	0.096	0.416	0.019

Note: Robust standard errors calculated using provincial clusters are listed below the coefficients. Exact *p*-values are shown in parentheses next to the coefficients. This table reports dynamic effects based on a PSM-DID matched sample. The matching procedure employed 1:1 nearest-neighbor matching based on baseline (2013) covariates.

**Table 6:** Summary of Results from Multiple Robustness Tests.

Variables	(1) ADL	(2) CES-D	(3) Ln *OOP*
Panel A: Excluding samples from municipalities directly under the central government			
Did (Excluding Municipalities)	−0.092 (*p* = 0.004)	−0.782 (*p* < 0.001)	−0.066 (*p* = 0.017)
	−0.029	−0.153	−0.026
Observed value (Obs)	45,732	45,732	45,732
			
Panel B: Hypothetical policy implementation date (assuming implementation in 2015)			
Did Fake (Placebo Timing)	0.045 (*p* = 0.051)	0.293 (*p* = 0.015)	−0.040 (*p* = 0.013)
	−0.022	−0.113	−0.015
Observed value (Obs)	33,849	33,849	33,849
			
Panel C: Excluding 2018, provinces that implemented the policy later			
Did (Excluding Late Imp.)	−0.077 (*p* = 0.003)	−0.803 (*p* < 0.001)	−0.048 (*p* = 0.087)
	−0.024	−0.163	−0.027
Observed value (Obs)	42,552	42,552	42,552
			
Panel D: PSM-DID			
Did (PSM-DID Model)	−0.082 (*p* = 0.011)	−0.620 (*p* < 0.001)	−0.037 (*p* = 0.213)
	−0.03	−0.146	−0.029
Observed value (Obs)	44,475	44,475	44,475
			
Panel E: Exclude household income as a control variable			
Did (Excluding Income)	−0.099 (*p* = 0.001)	−0.706 (*p* < 0.001)	−0.061 (*p* = 0.022)
	0.028	0.149	0.025
Observed value (Obs)	48,732	48,732	48,732
			
Control variables (Controls)	YES	YES	YES
FE	YES	YES	YES
Cluster SE	YES	YES	YES

Note: Robust standard errors clustered at the provincial level are reported below the coefficients. Asterisks denoting significance levels are not used. Panel A excludes data from the four municipalities directly under the central government (Beijing, Tianjin, Shanghai, Chongqing). Panel B retains only data from 2013 and 2015 for the placebo test. Panel C excludes provinces that completed the transition to the unified system as late as 2018.

**Table 7:** Mechanism Analysis: Pathways Between Healthcare Utilization and Financial Risk.

Variables	(1) Outpatient	(2) Inpatient	(3) CHE
Did (Treat × Post)	−0.016 (*p* = 0.121)	−0.004 (*p* = 0.326)	−0.019 (*p* = 0.004)
	−0.01	−0.004	−0.006
Treat	0.002	0.004	0.016
Post	−0.044	−0.049	−0.095
Control variables	YES	YES	YES
Fixed effects	YES	YES	YES
Observed Values (N)	48,732	48,732	48,732
$R^{2}$	0.021	0.026	0.044

Note: Robust standard errors clustered at the provincial level are reported below the coefficients.

**Table 8:** Heterogeneity Analysis: Regression by Education Level and Age (Ln *OOP*).

Variables	Panel A: SES		Panel B: Age	
	(1) Low-education group	(2) Higher education group	(3) Middle-aged group (45–74 years old)	(4) Elderly group (75 years and older)
Did (Treat × Post)	−0.046 (*p* = 0.356)	−0.044 (*p* = 0.167)	−0.067 (*p* = 0.024)	0.019 (*p* = 0.737)
	−0.049	−0.031	−0.028	−0.056
Control Variables and Fixed Effects	YES	YES	YES	YES
Observed Values (N)	23,362	25,370	42,905	5827
$R^{2}$	0.019	0.024	0.02	0.032

Note: Robust standard errors clustered at the provincial level are reported below the coefficients. Exact *p*-values are reported in brackets next to the coefficients.

## Data Availability

The data used in this study were drawn from the China Health and Retirement Longitudinal Study (CHARLS). CHARLS data are microdata available for public use; researchers can access them through the official website of the CHARLS Project Group at Peking University (http://charls.pku.edu.cn accessed on 1 September 2025).
